# Supplementary material for: The interactome of intact mitochondria by cross-linking mass spectrometry provides evidence for coexisting respiratory supercomplexes
Source: Mol Cell Proteomics. 2017 Dec 8;17(2):216–32. doi: 10.1074/mcp.RA117.000470 (PMC5795388; doi:10.1074/mcp.RA117.000470)
Supplement: Supplemental Data [file supp_RA117.000470_133922_0_supp_23978_fzffwf.zip › spectra_annotation/mito_spectra_annotation/273-1-7-1-2-1.pdf]

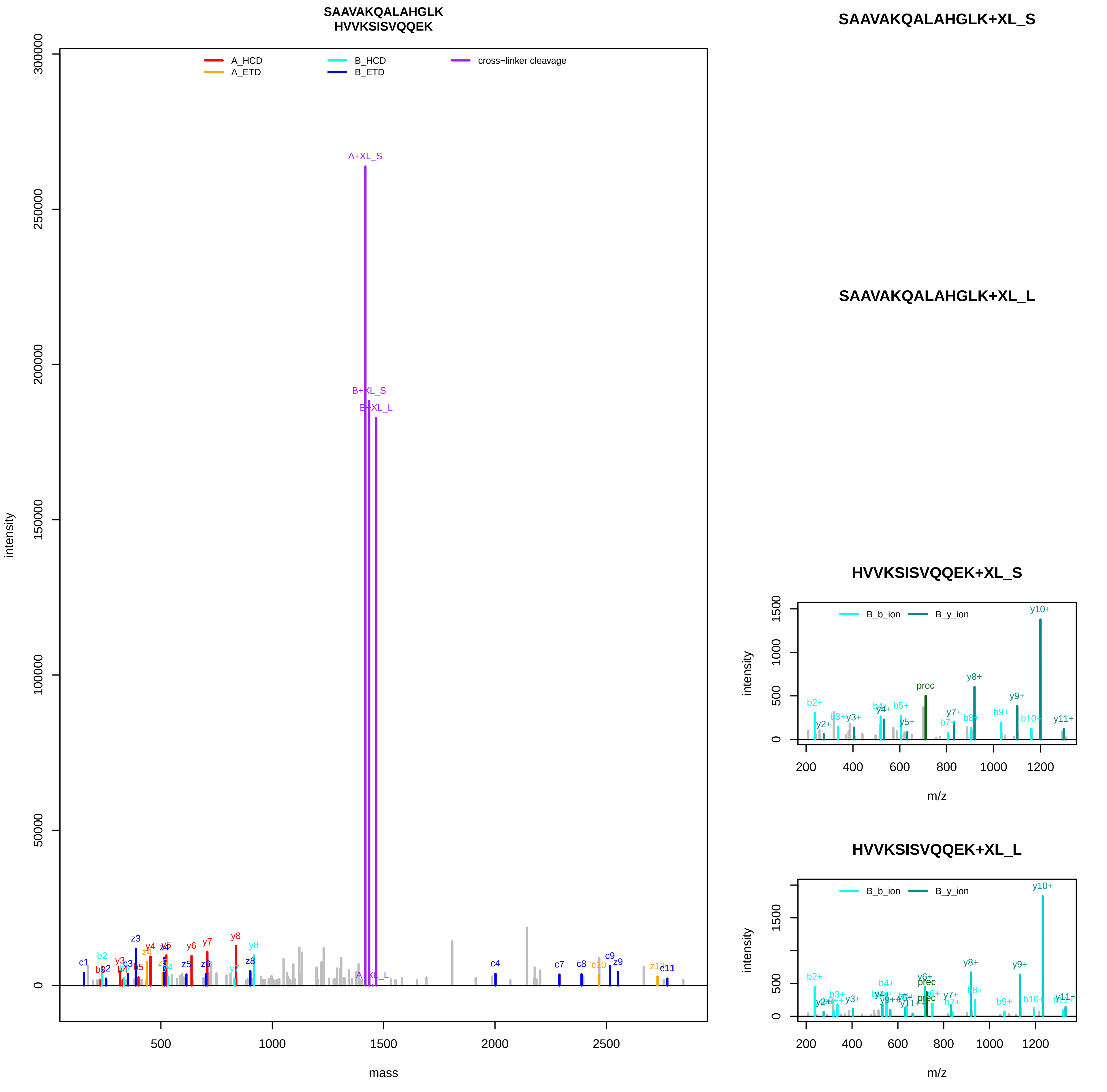
SAAVAKQALAHGLK
HVVKSISVQQEK
A_HCD
A_ETD
B_HCD
B_ETD
cross−linker cleavage
A+XL_S
B+XL_S
B+XL_L
A+XL_L
intensity
mass
0
50000
100000
150000
200000
250000
300000
500
1000
1500
2000
2500
c1
b2
b3
z2
y3
b4
c3
z3
b5
z4
y4
y5
z5
y4
z5
y6
z6
y7
y7
y8
z8
y8
c4
c7
c8
c10
c9
z9
c11
SAAVAKQALAHGLK+XL_S
SAAVAKQALAHGLK+XL_L
HVVKSISVQQEK+XL_S
B_b_ion
B_y_ion
intensity
m/z
prec
b2+
y2+
b3+
y3+
b4+
y4+
b5+
y5+
b7+
y7+
b8+
y8+
b9+
y9+
b10+
y10+
y11+
HVVKSISVQQEK+XL_L
b6+
y6+
b11+
y11+
200
400
600
800
1000
1200
0
500
1000
1500
